# A New Bolt Defect Identification Method Incorporating Attention Mechanism and Wide Residual Networks

**DOI:** 10.3390/s22197416

**Published:** 2022-09-29

**Authors:** Liangshuai Liu, Jianli Zhao, Ze Chen, Baijie Zhao, Yanpeng Ji

**Affiliations:** Electric Power Research Institute, State Grid Hebei Electric Power Co., Ltd., Shijiazhuang 050013, China

**Keywords:** deep learning, bolt defect recognition, wide residuals, double attention

## Abstract

Bolts are important components on transmission lines, and the timely detection and exclusion of their abnormal conditions are imperative to ensure the stable operation of transmission lines. To accurately identify bolt defects, we propose a bolt defect identification method incorporating an attention mechanism and wide residual networks. Firstly, the spatial dimension of the feature map is compressed by the spatial compression network to obtain the global features of the channel dimension and enhance the attention of the network to the vital information in a weighted way. After that, the enhanced feature map is decomposed into two one-dimensional feature vectors by embedding a cooperative attention mechanism to establish long-term dependencies in one spatial direction and preserve precise location information in the other direction. During this process, the prior knowledge of the bolts is utilized to help the network extract critical feature information more accurately, thus improving the accuracy of recognition. The test results show that the bolt recognition accuracy of this method is improved to 94.57% compared with that before embedding the attention mechanism, which verifies the validity of the proposed method.

## 1. Introduction

Bolts are the most numerous and widely distributed fasteners in transmission lines. As they play an important role in maintaining the stable operation of the lines, it is necessary to inspect the abnormal state of the bolts promptly so as to guarantee the safe and steady operation of the lines [1,2]. At present, the use of unmanned aerial vehicles (UAV) equipped with high-resolution cameras for transmission line inspection is not only safer and more efficient [3], but also can integrate deep learning-based image processing technology, which remarkably improves the quality and speed of inspection work. It is of great significance to study the bolted defect image recognition method based on deep learning.

Since the LeNet model was proposed, convolutional neural network models have shown considerable potential in image recognition tasks and have continued to develop. AlexNet [4] further increased the network depth and won the ImageNet challenge in 2012, and then ZFNet [5] and Google Inception Network (GoogLeNet) [6] were proposed one after another. Visual Geometry Group Network (VGGNet) [7] uses 16 convolutional layers and fully connects layers to improve the image recognition accuracy. However, the deepening of the network is not infinite. With the deepening of the number of network layers, problems caused by the deep network such as gradient disappearance and gradient explosion also emerge. The residual network (ResNet) proposed in [8] employs a jump connection method which effectively reduces the parameter number of the network, improves the training speed of the network, and ensures high accuracy. It is an effective solution to the problem that deep neural networks are difficult to train. Based on this, wide residual networks (WRNs) [9] further improve the model performance and increase the recognition accuracy by adding the number and width of convolutional layers to the residual blocks.

Currently, deep learning has been comprehensively used in bolt detection [10], defect classification [11], etc. In [12], the authors used multi-scale features extracted by cascade regions with a convolutional neural network (Cascade R-CNN) to build a path aggregation feature pyramid, which completes bolt defect identification. In [13], the authors enhanced the model complexity and improved the image recognition accuracy through the combined utilization of multiple algorithms. In [14], the authors used wide residuals as the backbone network and selected the optimal structure to achieve effective recognition of bolt defects by adjusting the network-widening dimension. In [15], a bolt defect data augmentation method was proposed based on random pasting, and it effectively expanded the number of bolt defect samples and improved the accuracy of defect recognition. However, due to the small size of the bolt itself, the bolt image features of the aerial transmission line are difficult to extract, and the bolt defect recognition effect is not satisfactory. The above method did not take into account the features of the bolt itself when improving the model.

The attention mechanism can help the network improve the feature extraction ability of the image [16,17]. It is a bionic of human vision that enables the acquisition of detailed information and the suppression of irrelevant information by allocating more attention to the target area. In the domain of deep learning, the attention mechanism uses the feature map to learn a new weight distribution, which is imposed on the original feature map. This weighting not only preserves the original information of the image extracted by the original network, but also enhances focus on the target region, effectively improving the performance of the model. The attention mechanism is not a complete network structure, but a plug-and-play lightweight module. When this module is embedded in the network, it can reasonably allocate computational resources and significantly increase the neural network performance at the cost of a finite increase in the number of parameters. Thus, it has received much attention in detection, segmentation, and recognition tasks because of its practicality and robustness [18,19,20]. Currently, it can be classified into three categories: spatial domain, channel domain, and hybrid domain. The squeeze and excitation attention network (SENet) [21] and efficient channel attention networks (ECA-Net) [22] are both of single-way attention frames that help the network detect or identify targets better by aggregating information in the spatial domain or channel domain and adaptively learning new weights. These networks are more concise than those with multi-way attention. The selective kernel network (SK-Net) [23] decomposes the feature map into feature vectors by decomposition, aggregation, and matching. In this way, the network is able to extract more detailed feature information. The convolutional block attention module (CBAM) [24] aggregates spatial and channel information to guide the model to focus on the key target regions in the image, while channel attention (CA) improves the ability to capture targets by aggregating one-dimensional channel and spatial information to relate the location relationships between targets in the feature graph. In [25], the authors proposed a dynamic supervised knowledge distillation method for bolt defect recognition and classification by applying knowledge distillation techniques to the bolt defect classification task and combining spatial channel attention. This method effectively improves the accuracy of bolt defect classification. In [26], the authors used an attention mechanism to locate the possible regions of the bolt in the image and then combined it with a deconvolutional network to build a model to achieve accurate detection of the bolt. This is an attention-based mechanism for transmission tower bolt detection. In [27], the authors embedded a dual-attention mechanism in faster regions with a convolutional neural network (Faster R-CNN) to analyze and enhance visual features at different scales and different locations, which effectively improved the bolt detection accuracy.

Although these methods improve the recognition or detection accuracy of bolts to some extent, they are all based on improving the feature expression capability of bolts without improving the model by combining bolt features. In order to identify bolt defects more accurately, by combining the attention mechanisms, we introduce bolt knowledge into the model and study the bolt defect recognition method incorporating dual attention in this paper. WRN is used as the backbone network, and the attention-wide residual network is designed by embedding squeeze and excitation networks [21] and coordinate attention [28] to enhance the network’s perception of features in the spatial dimension and channel dimension. The network was designed to enhance its ability to perceive features in the spatial dimension and channel dimension, extracting richer feature information. It is combined with the prior knowledge of bolts to achieve high-accuracy recognition of bolt defects.

## 2. Materials and Methods

**Figure 1:**
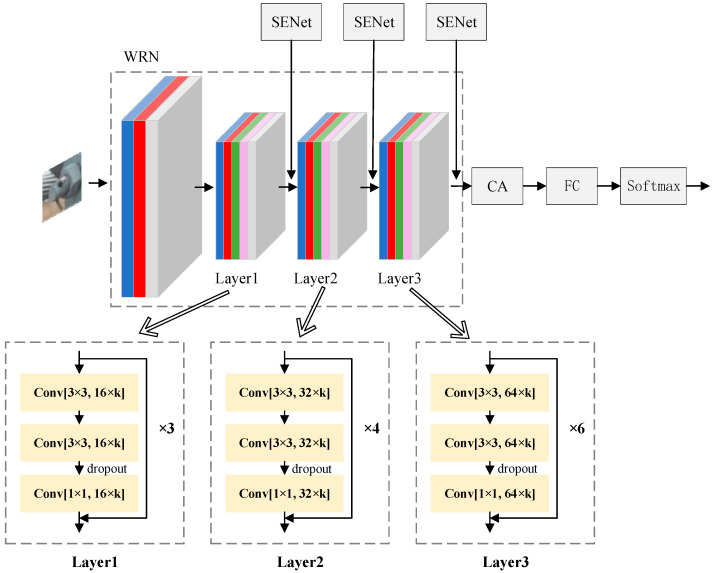
Attention to wide residual network structure.

In this work, WRN is used as the backbone network, and the number of channels is 16 × k, 32 × k, and 64 × k, a total of three levels. Among them, three wide residual blocks are in the first level, four wide residual blocks are in the second level, and six wide residual blocks are in the third level. The width factor k is taken as 2. The attention-wide residual network is designed by fusing the attention mechanism in the WRN, so as to enhance the extraction ability for bolt features and improve the accuracy of defect recognition. The overall structure is shown in Figure 1. Firstly, SENet attention is added to each level in the WRN to enhance the network’s ability to capture bolt defect features and output higher-quality feature maps. Secondly, CA attention based on structural prior knowledge is imported in combination with the spatial location relationship of pins and nuts on bolts, which enables the network to better utilize the feature location relationship and thus improve the accuracy of bolt defect recognition.

### 2.1. WRN Framework for Fusing Channel Attention

**Figure 2:**
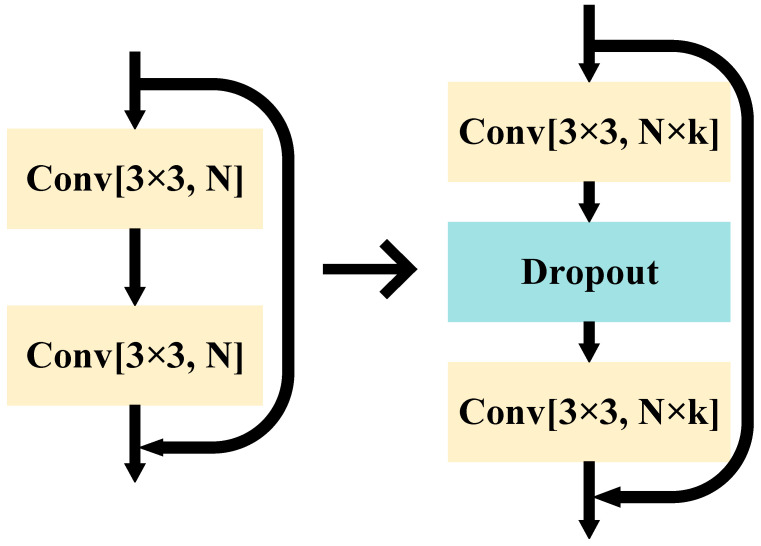
Schematic diagram of the relationship between ResNet block (**left**) and wide-ResNet block (**right**).

A residual network consists of a residual block. It is a constant mapping of shallow features to deeper features using a jump connection so that the residual block can learn more feature information based on the input features and effectively solve the degradation problem caused by deeper networks. However, as the number of network layers increases, the residual block itself cannot be better expressed. A new type of residual approach, WRN, which widens the number of convolutional kernels in the original residual block, was proposed. It effectively improves the utilization of the residual block, reduces the model parameters, speeds up the computation, and makes it possible to obtain a better training result without a deeper network layer. In addition, WRN adds a dropout between the convolutional layers in the residual block to form a wide ResNet block, which has the effect of improving the performance of the network. The relationship between the ResNet block and the wide ResNet block is shown in Figure 2, where 3 × 3 indicates the size of the convolution kernel, N is the number of channels, and k indicates the width factor.

SENet attention can aggregate the information from the input features at the spatial level and adaptively acquire new weight relationships through learning. These weight relationships represent the importance of different regions in the feature map, making the network focus on key regions in the feature map as a whole. It helps the information transfer in the network and continuously updates parameters in the direction that is beneficial to the recognition task.

**Figure 3:**
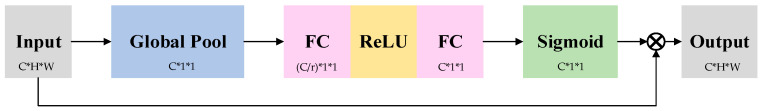
SENet attention structure diagram.

After fusing SENet attention in the WRN, the network first compresses the spatial dimension of the feature map of the input SENet through global average pooling, aggregating spatial information to perceive richer global features of the image and enhancing the network expression capability. The SENet attention structure diagram is shown in Figure 3. The global average pooling operation generates a feature map of C × 1 × 1 (where C represents the number of channels) to obtain the global information of channels. Then, the correlation between channels is captured by the two fully connected layers with the activation function of ReLu, and the normalized channel weights are then generated by the sigmoid activation function. At this point, the channel weights of dimension C × 1 × 1 can be multiplied with the input features of dimension C × H × W (where H represents the feature map of height, W represents the feature map of width) as a new parameter, i.e., the aligned channel dimension C. For each H × W matrix, a channel coefficient c is multiplied to obtain the output features C × H × W after SENet attention optimization, which enhances the key region features and suppresses irrelevant features to improve the performance of the network.

The attention weights are multiplied by the input features to obtain the output features *F*, as follows:

(1) $$F=\delta (MLP(Pool(F_{0})))\times F_{0}$$

where *F*_0_ denotes the input features, *δ* and *MLP* denote the sigmoid activation function and neural network operation, respectively, and *Pool* represents the pooling operation.

### 2.2. CA Attention with Integrated Knowledge

**Figure 4:**
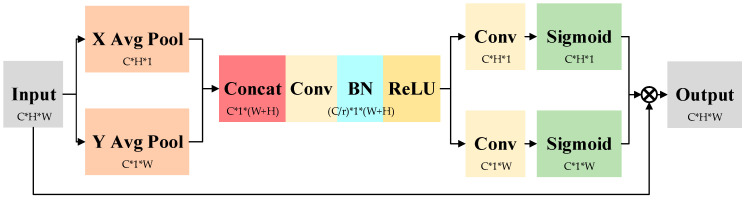
CA attention structure diagram.

The WRN incorporating SENet attention is enhanced to extract bolt features. However, according to the prior knowledge of the bolt, pins distribute at the head of the bolt while nuts usually locate at the root of the bolt, and these positional relationships are fixed. In order to further improve the bolt defect recognition accuracy using the bolt position information, we add CA attention to the output section of the WRN to enhance the positional relationships of the target. The CA attention structure is shown in Figure 4. First, CA attention decomposes the input features into a horizontal perceptual feature vector of dimension C × H × 1 and a vertical perceptual feature vector of dimension C × 1 × W by global averaging pooling in both directions. The one-dimensional feature vectors in the horizontal and vertical directions are as follows:

(2) $$z_{c}^{h}(h)=\frac{1}{W}\sum_{0\leq i<W}F_{c}(h,i)$$

(3) $$z_{c}^{w}(w)=\frac{1}{H}\sum_{0\leq j<H}F_{c}(j,w)$$

where *H* and *W* represent the height and width, respectively, *h*, *w*, *i*, and *j* represent the location coordinates in the feature map, *c* represents the number of channels, *z_c_^h^* represents the one-dimensional feature vector in the horizontal direction, *z_c_^w^* represents the one-dimensional feature vector in the vertical direction, and *F_c_* represents the input feature map.

In this process, the attention mechanism establishes long-term dependencies in one spatial direction and preserves precise location information in the other, helping the network locate key feature regions more accurately. It also gives the network a better global sensory view of the field as well as rich feature information. Next, the perceptual feature vectors in both directions are aggregated, and the feature mapping is obtained by dimensionality reduction through 1 × 1 convolution. Unique feature mappings are generated using two one-dimensional features.

(4) $$f=MLP([z^{h},z^{w}])$$

where [*z^h^*, *z^w^*] represents the stitching operation of two one-dimensional features, and *f* is the feature mapping of spatial information in the encoding process of horizontal and vertical directions. Finally, the feature mapping is decomposed and normalized by the Sigmoid function to obtain the attention weights in the two directions, and the attention weights in the two directions are multiplied with the input features of dimensionality C × H × W to obtain the output features of dimensionality C × H × W. The two directional weights and output features are as follows:

(5) $$g^{h}=\delta (T(f^{h}))$$

(6) $$g^{w}=\delta (T(f^{w}))$$

(7) $$F(i,j)=F_{c}(i,j)\times g_{c}^{h}(i)\times g_{c}^{w}(j)$$

where *T* represents the convolution operation and *F*(*i*, *j*) is the output feature. After the feature map is processed by CA attention, it is easier for the network to capture the key feature information in the map using location information, and the relationship between channels is more obvious.

## 3. Test Results and Analysis

### 3.1. Test Data and Settings

**Figure 5:**
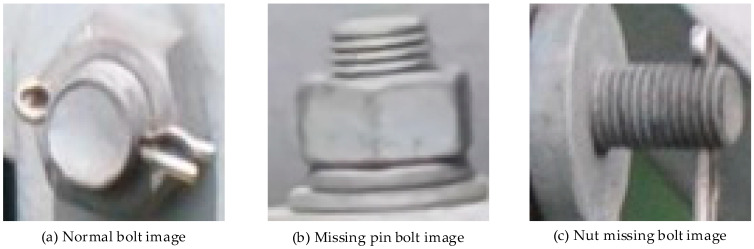
Three categories of bolt image samples.

Dataset Construction: We constructed a transmission line bolt defect recognition dataset by cropping and optimizing transmission line aerial images based on the Overhead Transmission Line Defect Classification Rules (for Trial Implementation). Tests were conducted to verify the effectiveness of this method. The dataset was divided into three categories, namely normal bolts, missing pin bolts, and missing nut bolts. There are a total of 6327 images, of which 2990 were normal bolts, 2802 were missing pin bolts, and 535 were missing nut bolts, and the training set and test set were divided in a ratio of 4:1. The samples of each category are shown in Figure 5.

Test Settings: The test hardware environment was Linux Ubuntu 16.04, and the GPU used is an NVIDIA GeForce 1080Ti with 11 GB of RAM. The test parameters were a batch size of 64, an epoch count of 200, and a learning rate of 0.1. We used the model to perform a recognition validation on the test set after the model completes an epoch training, obtain and save the accuracy and loss function values of the model on the test set, and take the highest recognition accuracy on the test set as the model evaluation metric after the model completes training. The accuracy rate was chosen as the evaluation index, and the formula is shown in Equation (8), where *TP* is the number of correctly predicted positive samples, *TN* is the number of correctly predicted negative samples, *FN* is the number of incorrectly predicted negative samples, and *FP* is the number of incorrectly predicted positive samples.

(8) $$Accuracy=\frac{TP}{TP+TN+FP+FN}$$

### 3.2. Ablation Tests and Analysis

In order to verify the effectiveness of this method in the actual bolt defect recognition task, we compared the accuracy of the test set under different methods by ablation experiments separately, as shown in Table 1. As can be seen, the recognition accuracy of the base model WRN was 93.31%, an improvement of 0.58% after adding SENet attention. This is because the SENet attention mechanism acquired richer bolt features by compressing spatial information, which enhanced the expressiveness of the network. With the addition of CA attention to the model, the attention mechanism builds long-term dependencies in space and the network is more likely to use the location relationships to capture key feature information, resulting in a 0.72% increment in recognition accuracy. The recognition accuracy of the model was improved by 1.26% after embedding both SENet attention and CA attention. The mutual association between the attentions further improved the network’s performance and it has accomplished a more accurate bolt defect recognition task.

**Figure 6:**
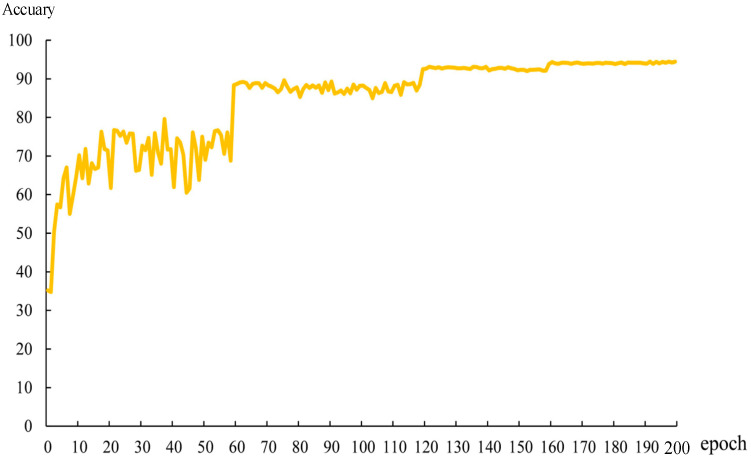
Accuracy curve on test set.

Figure 6 shows the variation curve of the recognition accuracy of the model on the test set as the number of training rounds increases. As can be seen, between epochs of 1 and 60, the accuracy of the model has the fastest rising trend, but the fluctuation is large, and the model has not learned efficient defect recognition ability. Between 60 and 120 epochs, the model’s learning task is initially completed, but the accuracy curve is still fluctuating. As the model was trained iteratively, the fluctuation of the accuracy curve gradually decreased after 120 epochs, and finally stabilized after 160 epochs.

**Figure 7:**
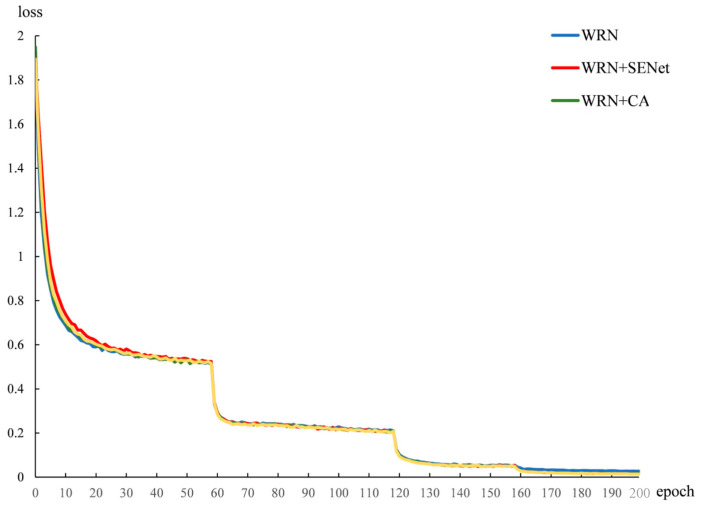
Convergence curve of the model training loss function.

Figure 7 shows the loss descent curves of different networks on the training set during the training process. As can be seen, the loss function convergence curves of the model training process under different approaches are compared. The first convergence was between epochs 1 and 60, during which the WRN model had the highest initial value, the WRN plus SENet had the slowest convergence, and the WRN plus CA attention had the fastest convergence. The second convergence was between epochs 60 and 120, and the third was between epochs 120 and 160. In these two convergence domains, the convergence rates and convergence trends of the four models were more or less the same, and the loss function convergence curves of each model showed slight fluctuations. The convergence trend of WRN is the weakest. WRN plus SENet and WRN plus CA attention are similar, and the convergence trend of our proposed method is the best.

**Figure 8:**
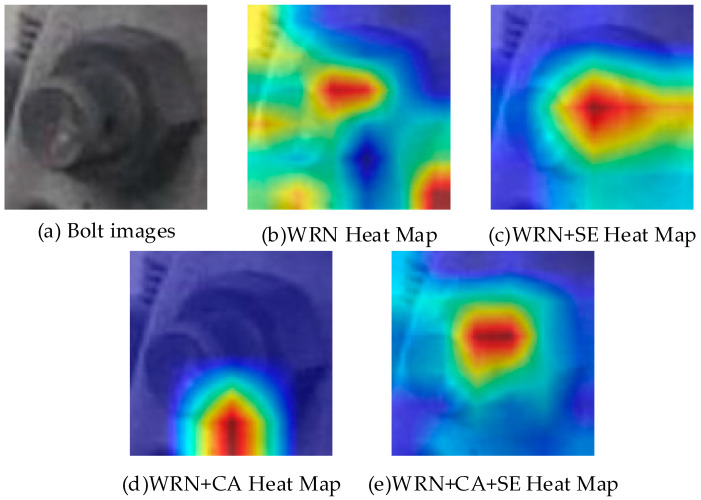
Visualization of the bolt feature map.

In order to demonstrate the improvement in model performance by attention more intuitively, we used the gradient-weighted class activation mapping (Grad-CAM) [29] algorithm to visualize the feature maps before and after the model improvement, as shown in Figure 8. In this test, a bolt image with missing pins was used as the reference. It can be seen from the figure that the attention area of the features extracted by WRN only is relatively scattered, which is not conducive to the recognition of the bolt by the model. Our method incorporates both SENet attention and CA attention, and the extracted feature map is more significant and discriminative compared with the previous ones. Our method effectively removes redundant information and allows the model to better distinguish bolt categories.

### 3.3. Comparative Tests and Analysis

In these tests, we compared the recognition accuracy of different recognition models for bolt defects in the test set, as shown in Table 2. WRN has the highest accuracy of 93.31%, 3.94% higher than VGG16, and 0.86% and 0.64% higher than ResNet50 and ResNet101, respectively. It fully demonstrates the feasibility and superiority of the backbone network selected in this paper, and paves the way for the next model improvement.

**Figure 9:**
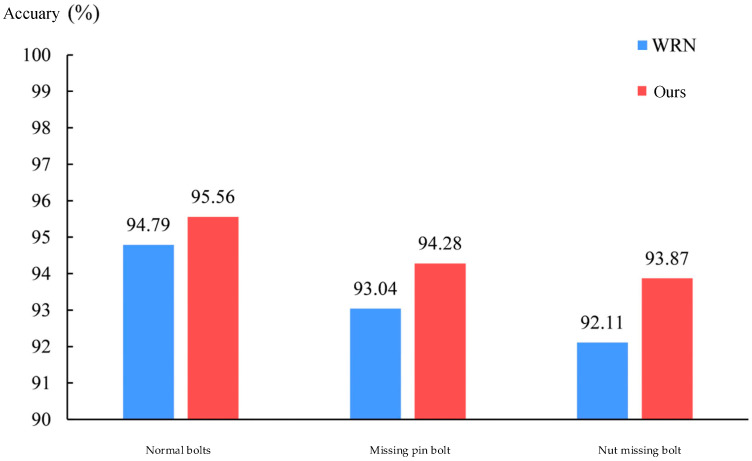
Comparison of classification accuracy before and after model improvement.

Meanwhile, we compared the recognition accuracy of each bolt before and after the improvement in the test set, as shown in Figure 9. As can be seen from the figure, after the improvement, the recognition accuracy was increased by 0.77% for normal bolts, 1.24% for missing pin bolts, and 1.76% for missing nut bolts. The accuracy improvement for normal bolts is less, while the accuracy improvement for bolts with missing pins and bolts with missing nuts is more significant with the help of the attention mechanism. This shows that the joint attention-wide residual method proposed in this paper is effective for bolt defect recognition. Embedding SENet attention into each layer to improve the ability of model feature extraction and combining CA attention to focus more accurately on the area of pin or nut in the figure helps the model to better discriminate the bolt category and improve the recognition accuracy.

## 4. Conclusions

In order to identify bolt defects more accurately, by taking WRN as the backbone network, we address the problem of difficult extraction of bolt features and the fixed position relationship of pins and nuts on top of the bolts. A new bolt defect identification method incorporating an attention mechanism and wide residual networks is proposed, embedding SENet and CA attention and fusing bolt knowledge. The proposed method can locate the key feature areas with better precision through collaborative space and channel information so as to help the model to improve the recognition accuracy. The proposed method has been validated on a homemade transmission line bolt defect recognition dataset. The test results show that the accuracy of this method was improved by 1.26% compared with that before improvement, which lays a foundation for the transmission line bolt defect detection task.

## Figures and Tables

**Table 1:** Ablation test results.

Method	Accuracy (%)
WRN	93.31
WRN + SENet	93.89
WRN + CA	94.03
Ours	94.57

**Table 2:** Ablation test results.

Recognition Model	Accuracy of Bolt Defect Recognition %
VGG16	89.37
ResNet50	92.45
ResNet101	92.67
WRN	93.31

## Data Availability

Not applicable.

## Abbreviations

The following abbreviations are used in this manuscript.

UAV	Unmanned Aerial Vehicle
GoogLeNet	Google Inception Network
VGGNet	Visual Geometry Group Network
ResNet	Residual Network
WRN	Wide Residual Networks
Cascade R-CNN	Cascade Regions with Convolutional Neural Network
SENet	Squeeze and Excitation Attention Network
ECA-Net	Efficient Channel Attention Networks
SK-Net	Selective Kernel Network
CBAM	Convolutional Block Attention Module
CA	Channel Attention
Faster R-CNN	Faster Regions with Convolutional Neural Network
Grad-CAM	Gradient-Weighted Class Activation Mapping
